# *OsTIP3;1* regulates rice seed germination via aleurone layer lipid metabolism: a transcriptome analysis

**DOI:** 10.3389/fpls.2026.1754622

**Published:** 2026-02-17

**Authors:** Yaping Cao, Yongkai Zhou, Heting Zhang, Huiping Chen

**Affiliations:** School of Life and Health Sciences, Hainan University, Haikou, China

**Keywords:** aleurone layer, lipid metabolism, *OsTIP3;1*, seed germination, transcriptome analysis, vacuolar fusion

## Abstract

Tonoplast intrinsic proteins (TIPs) are essential for water transport and cell physiology, however, their specific roles in seed development and germination, particularly in lipid metabolism, remain poorly understood. This study elucidates the role of rice *OsTIP3;1* using knockout (KN, *tip3;1-ii*) and overexpression (OE, OE-*TIP3;1-III*) lines. *OsTIP3;1* knockout led to significant defects in glume morphology and aleurone grain structure, whereas overexpression did not exhibit any adverse effects. During germination, KN seeds showed delayed radicle and plumule elongation, reduced lateral root development, lower germination energy (GE) and germination index (GI), although the final germination rate (GR) remained unchanged. In contrast, OE seeds displayed only transient inhibition of early radicle and plumule growth. Laser confocal microscopy (LCM) revealed asynchronous vacuolar fusion in aleurone cells, which was more pronounced in KN and OE lines than in the wild-type (WT). Transcriptome analysis identified distinct molecular pathways among genotypes. KN lines exhibited delayed fatty acid degradation, downregulated early-stage lipid metabolic genes, and enriched late-stage endoplasmic reticulum protein processing pathways, while OE lines showed enhanced vesicle transport processes. Protein-protein interaction network analysis identified lipid metabolism genes *LOC4335609* and *LOC4340986* as central hubs across all genotypes. Notably, RNA-seq and qRT-PCR analyses showed two genes were downregulated in KN lines at 1 d and 3 d of germination compared to the WT. These findings suggest that *OsTIP3;1* regulates rice seed germination potentially via modulating vacuolar fusion in the aleurone layer and coordinating lipid metabolic pathways to support efficient germination and seedling establishment.

## 1 Introduction

Rice (*Oryza sativa* L.) is a primary food source for the global population and a vital resource in agricultural production (Pickson et al., 2022). Rapid, uniform seed germination and vigorous seedling establishment are essential for achieving high and stable yields, as they are among the most critical stages in the rice life cycle (Gong et al., 2022; Li et al., 2022). The aleurone layer, situated between the starchy endosperm and the seed coat in cereal grains, plays a key role in seed germination (Guan et al., 2024) and supports embryonic growth through its multifunctional roles. During seed development and maturation, numerous granular structures, namely aleurone grains, accumulate and contain abundant storage reserves such as lipids, proteins, and minerals (Fath et al., 2000; Wang et al., 2007; Achary and Reddy, 2021). Lipids, a major category of rice nutrients, include triglycerides, phospholipids, fatty acids, and other bioactive components. They account for 2%-3% of grain weight, and are concentrated in the embryo and aleurone layer (Zhou et al., 2021). Their genetic and physiological roles in growth, stress responses, fertility, and seed longevity have been reviewed by Zhou et al. (2024). However, their metabolic links to seed germination remain poorly understood. Upon germination, the aleurone layer synthesizes and secretes hydrolytic enzymes (e.g., α-amylase) (Liang et al., 2025) that break down endosperm nutrients, thereby supporting embryonic development (Zheng et al., 2017). This process is coordinated with cellular remodeling, where organelle dynamics are critical.

Vacuoles, the central hubs for storage and degradation, undergo structural remodeling during seed development and germination. In plant cells, lytic vacuoles (LVs) and protein storage vacuoles (PSVs) undergo opposing patterns of fusion and fission (Zheng and Staehelin, 2011; Cui et al., 2020; Wu et al., 2024). A classic example is their dynamic role in stomatal opening and closure (Tanaka et al., 2007; Cao et al., 2022; Mirasole et al., 2023). In cereal aleurone layers, this remodeling is coupled to programmed cell death (PCD), a key event for successful germination (Xiao et al., 2021). During seed maturation, storage proteins are deposited in PSVs, which are called aleurone grains in the dry seed and become metabolically activated after imbibition (Robinson and Neuhaus, 2016). Subsequently, upon germination, these PSVs gradually fuse to form a large central vacuole, a process that initiates PCD, through subsequent vacuolar rupture, thereby mediating the death of aleurone cells to complete cereal seed germination (Zheng et al., 2017; Ashnest and Gendall, 2018; Zhang et al., 2020). This vacuolar transition triggers cellular reorganization, activates hydrolytic enzyme secretion, and enhances nutrient mobilization, thereby supporting embryonic growth and completing germination (Lee et al., 2015).

Aquaporins are integral membrane proteins that facilitate the transport of water and small solutes, functioning as essential channels, signaling hubs, and metabolic components in plants (Tyerman et al., 2021). They share a conserved hourglass-like topology with six transmembrane α-helices and two characteristic asparagine-proline-alanine (NPA) motifs on loops B and E that determine substrate selectivity (Kaldenhoff et al., 2007; Bezerra-Neto et al., 2019; Singh et al., 2020; Tang et al., 2023). Plant aquaporins are classified into several subfamilies (Maurel et al., 2016; Zhao et al., 2023; Rabeh et al., 2024), among which the tonoplast-intrinsic proteins (TIPs) are predominantly localized to the vacuolar membrane. TIPs, which are divided into five subgroups (TIP1-TIP5) in higher plants, play key roles in nutrient storage, turgor regulation, and stress adaptation (Sudhakaran et al., 2021; Zeng et al., 2024). Examples include *BpTIP1;3* enhancing drought tolerance in birch (Jia et al., 2025), *OsTIP1;2* conferring arsenite tolerance in yeast (Karle and Kumar, 2023), and *OsTIP2;1* involved in aluminum detoxification in rice (Zhang et al., 2024). Specifically, the TIP3 members exhibits a conserved, seed-specific expression pattern (Danielson and Johanson, 2008; Utsugi et al., 2015; Sudhakaran et al., 2024). The conservation is evident across species. For instance, in *Arabidopsis*, only *AtTIP3;1* and *AtTIP3;2* are detected in embryos during seed maturation and early germination (Gattolin et al., 2011). The functional importance of this conservation is demonstrated in soybean, where GmTIP3;1 and GmTIP3;2 transport H_2_O_2_ and boric acid, which are critical for seed germination (Sudhakaran et al., 2025). A pivotal finding in barley directly links *HvTIP3;1* to vacuolar remodeling: abscisic acid induces its expression to prevent PSVs coalescence, whereas gibberellin (GA) promotes the formation of a large central LV by reducing *HvTIP3;1* abundance (Lee et al., 2015, 2020). This highlights the role of *HvTIP3;1* in mediating hormone-dependent cellular reorganization during germination. However, whether its rice ortholog, *OsTIP3;1*, plays a similar regulatory role in the aleurone layer remains unknown. Moreover, the downstream molecular pathways through which *OsTIP3;1* coordinates vacuolar dynamics, nutrient metabolism, and germination traits are yet to be elucidated.

Therefore, the research used *OsTIP3;1* knockout and overexpression lines to systematically investigate its effects on rice seed and seedling morphology, germination characteristics, dynamic changes in aleurone cell structure, and the underlying molecular mechanisms via RNA-seq. The study demonstrates that *OsTIP3;1* is a key regulator of rice seed germination. The integrated phenotypic, cellular, and molecular evidence supports a model whereby *OsTIP3;1* modulates vacuolar fusion and lipid metabolism to coordinate germination, providing novel insights into the regulatory network of cereal seed germination.

## 2 Materials and methods

### 2.1 Plant materials and culture

Wild-type (WT), *OsTIP3;1*-knockout (KN, *tip3;1-ii*), and *OsTIP3;1*-overexpression (OE, OE-*TIP3;1-III*) rice lines, generated and preserved in our laboratory, were used in this experiment. The *OsTIP3;1* knockout lines were generated via CRISPR-Cas9 technology. Two sgRNAs targeting the exonic regions of *OsTIP3;1* were designed and cloned into the binary CRISPR vector *pYLCRISPR/Cas9Pubi-H*. For overexpression analysis, the full-length coding sequence of *OsTIP3;1* was amplified from cDNA of *Oryza sativa* ssp. japonica cv. Nipponbare and cloned into the *PU1300* vector, under the control of the maize Ubiquitin promoter. The resulting construct was introduced into *Agrobacterium tumefaciens* strain EHA105 and delivered into rice via callus-mediated stable transformation. Homozygous transgenic lines from the T_2_ generation or beyond were used for all experiments to ensure genetic uniformity and stable transgene expression.

Dry rice grains were dehusked, and plump, viable seeds were selected. The seeds were surface sterilized with 0.1% KMnO_4_ for 10 min, rinsed three times with distilled water, and then placed on petri dishes containing distilled water for germination. The seeds were germinated in a light incubator at 25 °C under a 16 h/8 h (light/dark) photoperiod with a light intensity of 3,000 lux for 7 d.

### 2.2 Seed phenotypic observation and germination assessment

Glumes and mature seeds were harvested separately from the WT, *tip3;1-ii*, and OE-*TIP3;1-III* lines at both the heading and maturity stages. Their morphology were observed under a stereomicroscope (Olympus, SZ61). For these lines, the determined germination characteristics included seed germination rate (GR), germination energy (GE), germination index (GI, normalized), radicle length, embryo length, number of lateral roots, and lateral root length. The radicle is the embryonic root emerging during seed germination, and the radicle length refers to its linear measurement from base to tip. Radicle length measured with a ruler at 3–7 d of germination (lateral roots were excluded from radicle length assessments). A seed was considered germinated when radicle emergence reached ≥ 2 mm. The calculation formulas are as follows:


$$\text{GE}=\frac{\text{number of seeds germinated on the }3\text{rd day}}{\text{number of seeds tested}}\times 100\%$$

$$\text{GR}=\frac{\text{number of seeds germinated on the }7\text{th day }}{\text{number of seeds tested}}\times 100\%$$

$$\text{GI}=\sum \frac{G_{t}}{D_{t}}$$

Where G_t_ represents the number of seeds germinated on day t and D_t_ denotes the corresponding day number. Data were statistically analyzed using IBM SPSS Statistics (version 24.0). Results are presented as mean ± SD from five independent replicates. Normality and homogeneity of variance were assessed. Data meeting parametric assumptions were analyzed using one-way ANOVA followed by Duncan’s test. For data not meeting these assumptions, the Kruskal-Wallis H test was employed, with significant results followed by pairwise comparisons (Mann−Whitney U test, P < 0.05). Figures were prepared using GraphPad Prism (version 9.4.1).

### 2.3 Observation of aleurone grain morphology using scanning electron microscopy

Dry, intact rice grains were dehusked as previously described (Demone et al., 2022). Then, the pipette tip assemblage was used to fasten the seed, and a scalpel was employed to cut it into 1 mm thick cross-sections, with five biological replicates per group. Finally, the prepared sections were fixed on conductive adhesive, a uniform platinum conductive film was then sputtered onto their surface using an ion sputter coater (JFC-1600, Japan) for 30 s. Aleurone cell morphology was observed under a field emission scanning electron microscope (JSM-7100F, Japan) at 5 kV and 5000× magnification, with three random fields captured per sample.

### 2.4 Observation of aleurone cells using a laser confocal microscope

Aleurone layers were stripped from WT, *tip3;1-ii*, and OE-*TIP3;1-III* seeds at 1 d, 3 d, 5 d, and 7 d of germination, respectively. The outer pericarp and seed coat tissues were carefully dissected away using a scalpel, after which the aleurone layers were cut into small fragments and fixed in FAA for 30 min. Subsequently, aleurone fragments were permeabilized in 0.1% BSA/Triton X-100 buffer (3 cycles, 5 min each). Each cycle was followed by a PBS rinse, and two additional PBS rinses were performed after the final cycle. After slide mounting, aleurone cells were visualized using a LCM.

### 2.5 RNA extraction, transcriptome sequencing, and qRT-PCR validation

Aleurone layers were stripped from seeds of WT, *tip3;1-ii*, and OE-*TIP3;1-III* at 1 d, 3 d, 5 d and 7 d of germination, with three biological replicates per group. Total RNA was extracted from aleurone layer using the RNAprep Pure Plant Kit (DP441, Tiangen, China). RNA purity (assessed via Nanodrop 2000) and integrity (evaluated using Agilent 2100, RIN ≥ 5.0) met the quality requirements for downstream analyses. For each sample, 1 μg of total RNA was used for library construction. Raw FASTQ data were filtered, and quality metrics including Q20, Q30, GC content, and sequence duplication levels were calculated. Clean reads were generated by filtering adapter-containing and low-quality reads from the raw data. These clean reads were then aligned to the reference genome of *O. sativa* L. ssp. japonica (GWH Accession No.: ASM3414082v1) using HISAT2. StringTie was employed to splice and assemble the mapped reads, and functional annotation was performed against databases including GO (Gene Ontology), KEGG (Kyoto Encyclopedia of Genes and Genomes), and NR (NCBI non-redundant protein sequences) (Zhang et al., 2025).

Quantitative Real-Time PCR (qRT-PCR) was performed to validate the gene expression patterns identified by RNA-seq analysis. Total RNA (1 μg, qualified) was reverse-transcribed into first-strand cDNA using a PrimeScript™ RT reagent Kit with gDNA Eraser (TaKaRa, RR047A, Japan) according to the manufacturer’s instructions. Gene-specific primers (**Supplementary Data Table S1**) for target genes (*LOC4335609* and *LOC4340986*) and reference genes (*18S rRNA* and *Actin*) were designed using Primer Premier 5.0 software and synthesized by Sangon Biotech (Shanghai, China). The qRT-PCR reactions were performed on a LightCycler^®^ 480 Real-Time PCR System (Roche, Switzerland) using TB Green^®^ Premix Ex Taq™ II (TaKaRa, Japan). The 20 μL reaction system contained 10 μL of 2× TB Green^®^ Premix Ex Taq™ II, 0.6 μL of each primer (10 μmol/L), 1 μL of cDNA template, and 7.8 μL of RNase-free H_2_O. Amplification program was carried out under the following conditions: preincubation at 95 °C for 60 s, followed by 40 cycles of denaturation at 95 °C for 5 s, annealing at 60 °C for 15 s, and extension at 72 °C for 15 s. Melting curve analysis was performed post-amplification to verify primer specificity. The relative expression levels of target genes were calculated using the 2^⁻ΔΔCt^ method, with *18S rRNA* and *Actin* serving as dual internal controls. All qRT-PCR assays were included three biological replicates.

### 2.6 Transcriptome analysis

Gene expression levels were quantified as FPKM (Fragments Per Kilobase of transcript per Million mapped reads). Differentially expressed genes (DEGs) were identified via DESeq2, with screening thresholds set as log_2_FC (Fold Change) ≥ 2 and adjusted *p*-value< 0.01. For DEGs derived from the WT, KN, and OE lines, GO and KEGG enrichment analyses were conducted. The GO terms encompassed three categories: biological process (BP), cellular component (CC), and molecular function (MF). KEGG pathway enrichment analysis was performed using KOBAS and clusterProfiler, where a *q*-value< 0.05 was considered statistically significant. DEGs from WT, KN, and OE lines were clustered using Mfuzz to identify co-expressed gene modules and their enriched functional pathways. Heatmaps was generated using TBtools software. The Protein-Protein Interaction (PPI) network of DEGs was constructed using Cytoscape software (v.3.9.1). The top 20 hub genes were screened based on degree algorithm and analyzed via the cytoHubba plugin; the visualization of related graphs was primarily implemented using platforms or software based on R or Python packages.

## 3 Results

### 3.1 Morphological analysis of seed phenotype and aleurone grain structure

**Figure 1:**
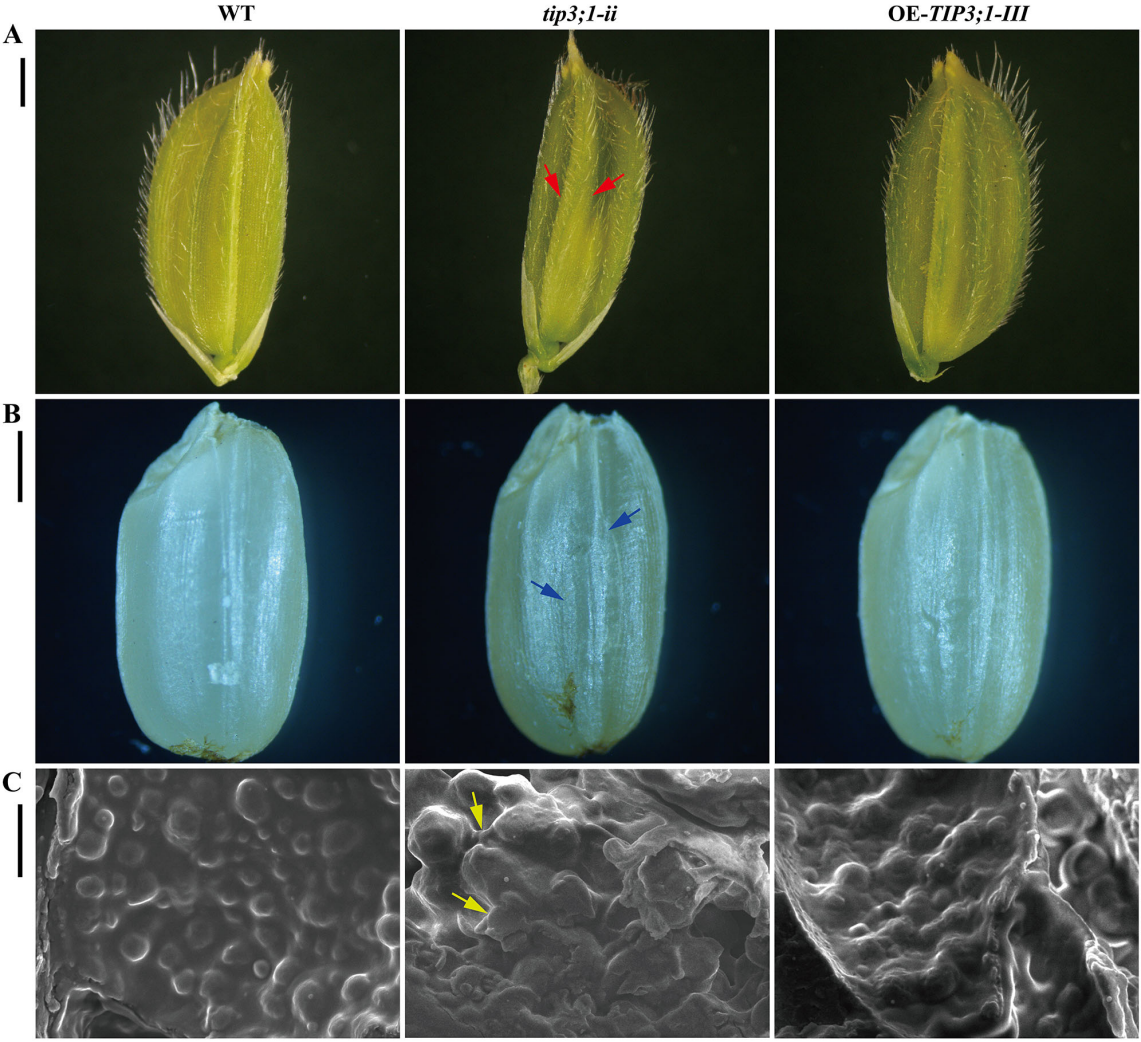
Comparison of seed morphology among wild-type (WT), *tip3;1-ii* mutant and OE-*TIP3;1-III* overexpressed rice plants. **(A)** Morphological diagram of rice glumes. Red arrows indicate the protruding, irregular ridge-like structure in the *tip3;1-ii* mutant (scale bar = 1 mm). **(B)** Morphological image of mature rice seeds. Blue arrows indicate the ridged and concave surface in the *tip3;1-ii* mutant (scale bar = 100 μm). **(C)** 5000× magnification view of the aleurone layer region in dry rice seed cross−section. Yellow arrows indicate the location where aleurone grains interconnected in the *tip3;1-ii* mutant (scale bar = 5 μm).

To investigate the role of the *OsTIP3;1* gene in rice seed development, we first observed glume phenotypes at the heading stage and mature seed morphologies, then analyzed the microstructure of aleurone grains in dry mature seeds across WT, *tip3;1-ii*, and OE-*TIP3;1-III* lines. The glume phenotypes and mature seed morphologies of the three lines are presented in **Figures 1A, B**. Compared to the WT, the glumes of *tip3;1-ii* mutant showed distinct abnormal characteristics. Its surface showed a visible irregular ridge-like structure under stereomicroscopy (**Figure 1A**, *tip3;1-ii*, red arrows), and it was significantly narrowed in width. The glume defects further result in mature seed with a ridged and concave surface (**Figure 1B**, *tip3;1-ii*, blue arrows). The phenotypic abnormalities suggest that knockout of the *OsTIP3;1* gene disrupts critical developmental processes during glume formation, such as coordinated cell differentiation and cell expansion, and that glume malformation contributes to the irregular (ridged and concave) surface of mature seeds. In contrast, the glumes of the OE-*TIP3;1-III* lines showed no detectable differences from the WT in terms of surface texture, shape, or size. Similarly, mature seeds of the OE lines were indistinguishable from WT seeds in length, width, and plumpness. This indicates that overexpression of *OsTIP3;1* does not interfere with normal rice seed development, even when the gene is constitutively expressed.

To further explore cellular level defects caused by *OsTIP3;1* knockout, we observed the microstructure of aleurone grains in the aleurone layer of dry mature seeds using scanning electron microscopy (**Figure 1C**). In the WT lines, aleurone grains displayed a relatively regular spherical or oval morphology, with intact and smooth outer membranes. Individual grains were clearly separated, and their overall arrangement within the aleurone layer was uniform. By contrast, the *tip3;1-ii* showed striking and heterogeneous alterations in aleurone grain structure. The grain surfaces exhibited severe structural disruptions, including deep folds, irregular protrusions, and localized depressions, likely resulting from impaired membrane integrity. Additionally, multiple aleurone grains aggregated into clumps, failing to maintain their individual morphological identity (**Figure 1C**, *tip3;1-ii*, yellow arrows). These results confirm that loss of *OsTIP3;1* gene function leads to abnormal aleurone grain development at the cellular level, which may compromise the aleurone layer’s ability to support seed germination. In the OE-*TIP3;1-III* lines, aleurone grains maintained their individual spherical shape, but their surfaces were covered with small vesicle-like structures (**Figure 1C**, OE-*TIP3;1-III*). These vesicles may be associated with enhanced vesicular trafficking, however this minor structural modification did not compromise the overall integrity or individual identity of the aleurone grains.

### 3.2 Dynamic changes of vacuoles in aleurone cells during germination

**Figure 2:**
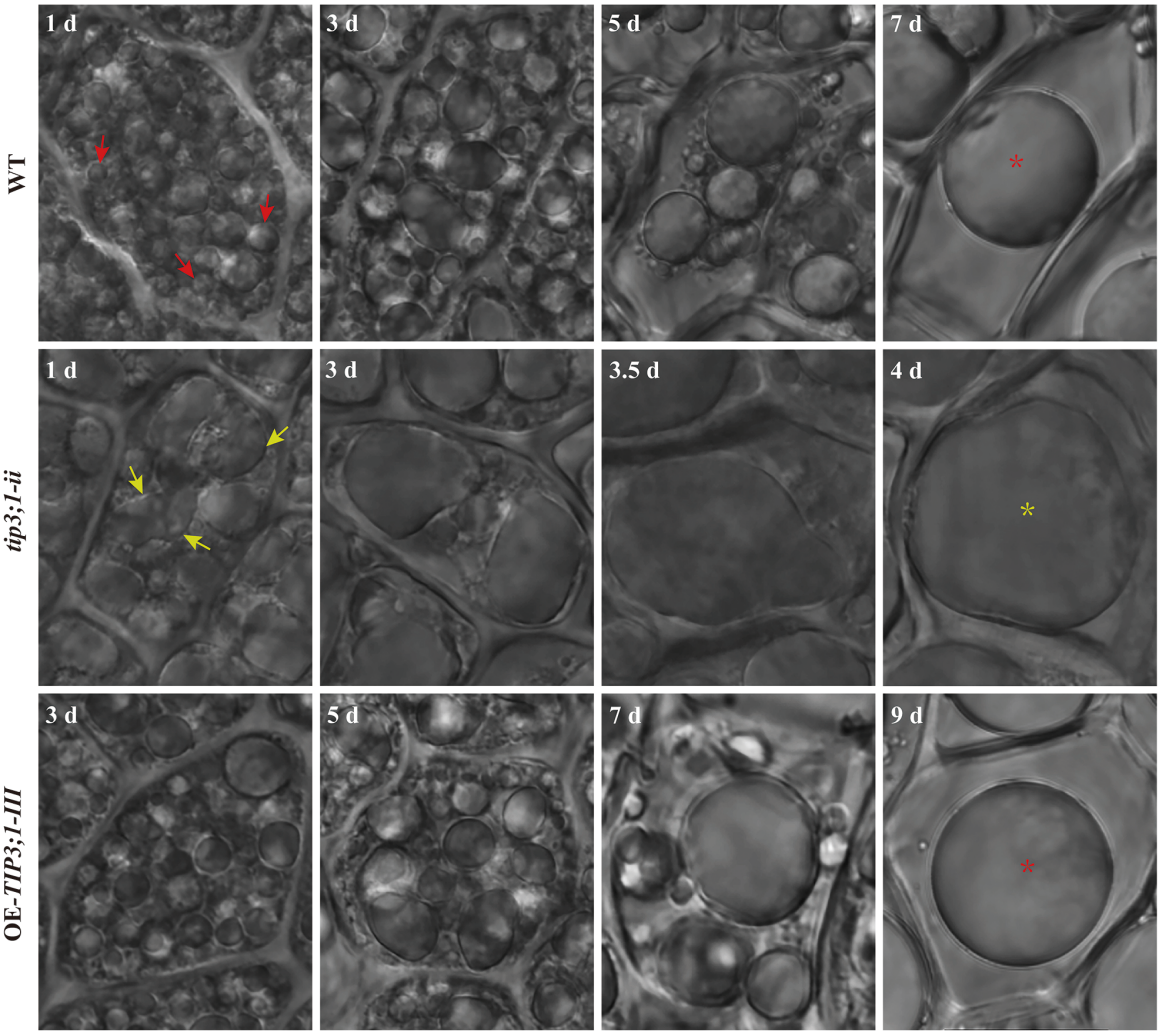
Morphological characteristics of contents in aleurone cells during seed germination of wild-type (WT), *tip3;1-ii* mutant and OE-*TIP3;1-III* overexpressed rice plants. Top row: Aleurone vacuole morphologies of WT at 1 d, 3 d, 5 d and 7 d of germination. Red arrows indicate numerous dense vacuoles at 1 d, and red asterisk indicates a large central vacuole at 7 d. Middle row: Aleurone vacuole morphologies of *tip3;1-ii* mutant at 1 d, 3 d, 3.5 d and 4 d of germination. Yellow arrows indicate multiple irregular larger vacuoles at 1 d, and asterisk indicates the large central vacuole on the verge of rupture at 4d. Bottom row: Aleurone vacuole morphologies of seeds from OE-*TIP3;1-III* overexpressed lines at 3 d, 5 d, 7 d and 9 d of germination. Red asterisk indicates the large central vacuole formed at 9 d. Scale bars in all panels = 20 μm.

To investigate the cellular mechanisms underlying genotypic differences in seed germination, we employed LCM to observe aleurone cells in WT, *tip3;1-ii*, and OE-*TIP3;1-III* lines, with a focus on this critical nutrient-mobilizing tissue. In WT seeds (**Figure 2**, the top row), the aleurone cells were filled with numerous dense vacuoles at 1 d (**Figure 2**, red arrows of WT-1 d). As germination progressed, the number of these small vacuoles gradually decreased, while their volume gradually increased. At 7 d, a large, spherical central vacuole had formed (**Figure 2**, red asterisk of WT-7d). In *tip3;1-ii* seeds (**Figure 2**, the middle row), multiple irregular larger vacuoles appeared at 1 d (**Figure 2**, yellow arrows of *tip3;1-ii*-1d). At 4 d of germination, the large central vacuole was observed to be on the verge of rupture (**Figure 2**, yellow asterisk of *tip3;1-ii*-4d), presumably due to rapid fusion events in the mutant. In OE-*TIP3;1-III* seeds (**Figure 2**, the bottom row), the aggregation process was slower than that in WT. At 3 d, the morphology of its vacuoles was nearly identical to that of WT at 1 d. Unexpectedly, a large central vacuole was formed only until 9 d of germination (**Figure 2**, red asterisk of OE- *TIP3;1-III*. -7d).

The above results indicate that there is a significant mutual fusion trend of vacuoles in aleurone cells during seed germination. However, the timing of this fusion and the formation of central vacuoles differed significantly among WT, *tip3;1-ii*, and *OE-TIP3;1-III*. It remains unclear whether this asynchronous vacuolar fusion is a direct contributor to the genotypic differences in seed germination documented in our earlier work, and additional studies are required to clarify this relationship.

### 3.3 Effects of *OsTIP3;1* on seed germination characteristics

**Figure 3:**
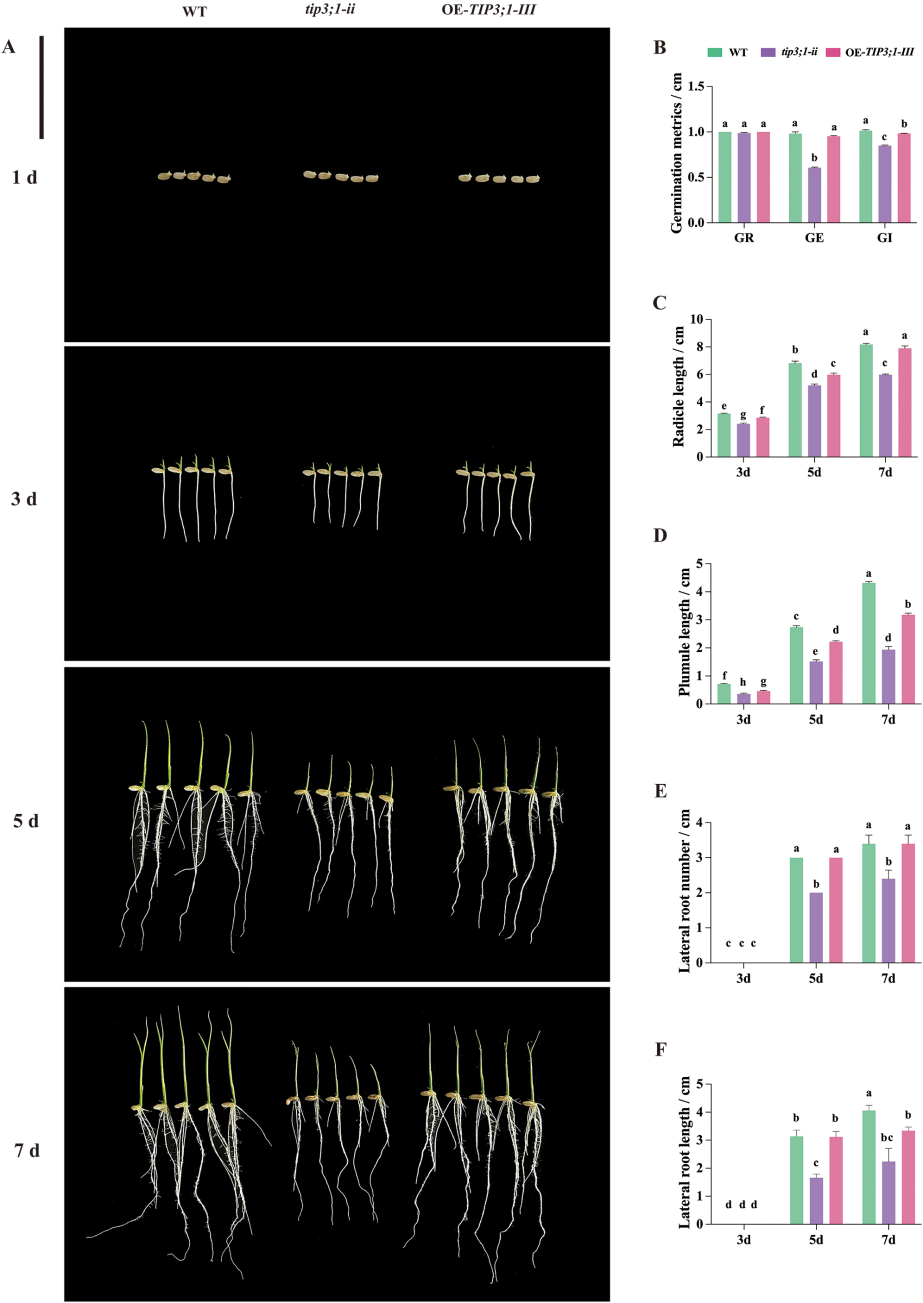
Germination morphology and related index statistics of seeds from wild-type (WT), *tip3;1-ii* mutant and OE-*TIP3;1-III* overexpressed rice plants. **(A)** Seed germination morphology (scale bar = 5 cm). **(B)** Showed the statistical results of germination rate (GR), germination energy (GE) and germination index (GI) during seed germination. **(C–F)** Showed the radicle length **(C)**, plumule length **(D)**, lateral root number **(E)** and lateral root length **(F)** at different germination time. Different lowercase letters in panels B–F indicate statistically significant differences at the *P* < 0.05 level; bars with the same letter are not significantly different.

To investigate the role of the *OsTIP3;1* gene in seed germination, phenotypic characteristics of WT, *tip3;1-ii*, and OE-*TIP3;1-III* seeds were observed at 1 d, 3 d, 5 d, and 7 d of germination (**Figure 3A**), and germination traits (including GR, GE, and GI) were further analyzed (**Figure 3B**). The *tip3;1-ii* lines had a slightly lower GR (96.67%) but remained high, while the GR of WT and OE-*TIP3;1-III* lines reached 100%. These results demonstrate that neither *OsTIP3;1* knockout nor overexpression affects GR. At 3 d of germination, the WT lines exhibited significantly higher GE and GI than the *tip3;1-ii* lines did, whereas the OE-*TIP3;1-III* lines did not differ significantly from the WT. These findings suggest that *OsTIP3;1* is a key regulatory factor for early rapid and uniform germination in rice seeds, as its knockout delays the germination (reflected by reduced GE and GI). Overexpression of the *OsTIP3;1* gene failed to further accelerate seed germination speed, possibly due to functional redundancy.

Additionally, plumule length, embryo length, number of lateral roots, and lateral root length were quantified (**Figures 3C–F**). The results showed that the radicle lengths of seedlings among the lines significant differences over time. At 3 d and 5 d, WT radicles were significantly longer than those of OE-*TIP3;1-III* and *tip3;1-ii*. However, at 7 d, radicle length did not differ significantly between OE-*TIP3;1-III* and WT. These findings indicate that knockdown of *OsTIP3;1* has a persistent inhibitory effect on radicle elongation during seed germination, whereas the inhibitory effect of *OsTIP3;1* overexpression on radicle growth is mainly observed from 1 d to 5 d, and diminishes at 7 d. At 3 d, 5 d, and 7 d, the plumules of WT were significantly longer than those of OE-*TIP3;1-III* and *tip3;1-ii*, with *tip3;1-ii* exhibiting the shortest plumules. Simultaneously, lateral root development had not yet initiated in any line at 3 d of germination. At 5 d and 7 d, both the number and length of lateral roots in WT and OE-*TIP3;1-III* were significantly greater than those in *tip3;1-ii*.

Taken together, *OsTIP3;1* plays a critical regulatory role in the development of radicles, plumules, and lateral roots in rice seedlings. Knockout of *OsTIP3;1* generally inhibits the growth of these structures, resulting in significantly reduced trait values compared to WT. Overexpression of *OsTIP3;1* also inhibits radicle and plumule growth but exerts weaker effect on lateral roots. Regarding germination traits, *OsTIP3;1* exerts minimal effect on final GR but strongly regulates early germination speed, with knockout mutants showing higher GRs but delayed speed.

### 3.4 Comprehensive evaluation of RNA-seq data quality with clustering analysis

To further explore the molecular mechanisms underlying the differences in aleurone cell vacuolar fusion and seeds germination among WT, *OsTIP3;1*-knockout (KN) and *OsTIP3;1*-overexpression (OE) lines, we built on earlier morphological observations and conducted transcriptome sequencing. For the experiment, three biological replicates were designed for each rice genotype (WT, KN, OE) and each time point (1 d, 3 d, 5 d, 7 d of germination). RNA was isolated from the aleurone layer of the three genotypes at the specified time points. A total of 36 RNA samples were sequenced on the Illumina platform. Key sequencing quality metrics were as follows: the Clean Data for each sample reached 5.71 Gb, with Q20 probability above 98.30%, and Q30 probability exceeding 95.1%. The GC content ranged from 50.92% to 54.25% (**Supplementary Data Table S2**). These results confirm the high quality of the sequencing data, and the sequencing results meet the analysis requirements. The *O. sativa* japonica reference genome (GWH Accession: ASM3414082v1) was used for sequence assembly and annotation (**Supplementary Data Table S3**). Clean reads of all samples showed 88.73%-97.99% alignment to the reference genome, including 91.41%-94.50% unique mappings and 2.28%-2.33% multi-mappings. This high alignment rate confirms the data is suitable for gene expression analysis.

**Figure 4:**
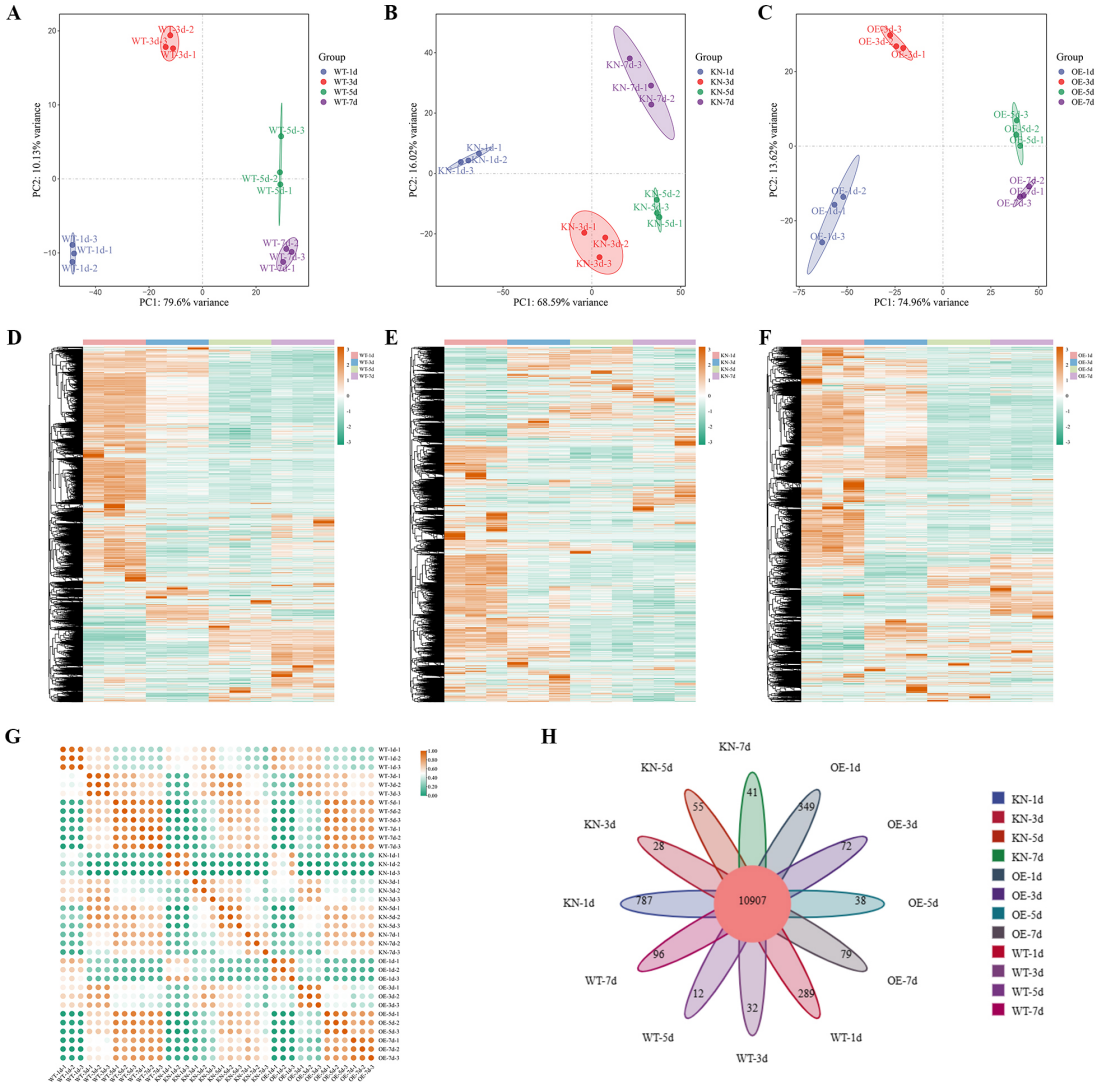
Heatmap of inter-sample expression correlation. **(A–C)** Principal component analysis (PCA) plots, for WT **(A)**, KN **(B)**, and OE **(C)** samples. The coordinates represent principal components, with percentages indicating their contribution to sample variance. Each point represents an individual sample, and groups are distinguished by color and shape. **(D–F)** Clustering heatmap of genes expression in WT **(D)**, KN **(E)**, and OE **(F)** samples. **(G)** Inter-sample expression correlation heatmap. The x-axis and y-axis represent sample identifiers, and color intensity reflects correlation strength (higher values = stronger correlation). **(H)** Number of genes detected per sample (bar plot).

This study conducted Principal Component Analysis (PCA) and cluster analysis on 36 aleurone layer samples. For the WT (**Figure 4A**), PC1 explained 79.60% of the variance, with samples from different time points and replicates clustering tightly (low within-group variation). For the OE (**Figure 4B**), PC1 accounted for 74.96% of the variance, while the KN’s PC1 (**Figure 4C**) explained 68.90%. Both OE and KN lines showed higher sample dispersion but clear inter-group separation. The cluster heatmap results of genes in WT, KN, and OE samples (**Figures 4D-F**) showed differences in gene expression profiles among these genotypes, with the gene expression pattern in KN samples being obviously, different from that in WT and OE samples. This implied that the knockout of the *OsTIP3;1* gene has a substantial impact on the transcriptome, leading to altered expression of multiple genes. The WT samples showed high diagonal correlation (**Figure 4G**, deep red), indicating strong transcriptome similarity, whereas the OE and KN lines exhibited weaker diagonal correlations. The low off-diagonal correlations between WT-OE/KN and OE-KN reflected significant regulatory pathway differences. A total of 12,785 genes were annotated across WT, OE, and KN, with 10,907 core genes (85.30% of total) shared among all lines. These shared genes are involved in core metabolic pathways common to the three genotypes (**Figure 4H**).

### 3.5 GO enrichment analysis of DEGs

**Figure 5:**
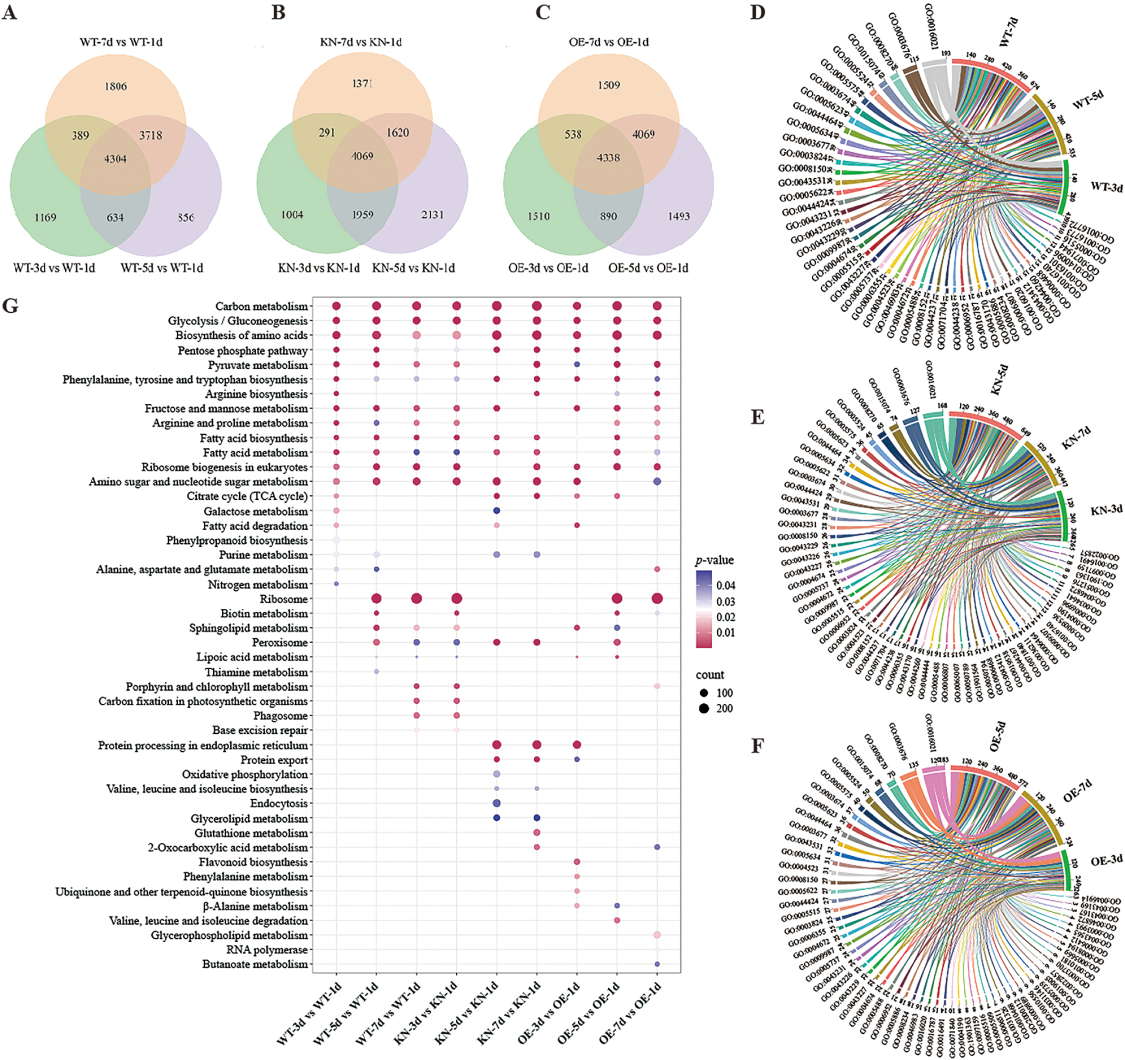
Functional annotation analysis. **(A–C)** Venn diagram of DEGs for WT **(A)**, KN **(B)**, and OE **(C)** groups. **(D-E)** GO functional annotation analysis of DEGs in WT **(D)**, KN **(E)**, and OE **(F)** groups. **(G)** KEGG enrichment bubble map of DEGs in WT, KN and OE groups.

Venn diagrams results showed that compared with the control at 1 d, WT, KN, and OE had 4,304, 4,069, and 4,338 shared DEGs, respectively, clearly illustrating the distribution of DEGs at the four time points (1 d, 3 d, 5 d, and 7 d of germination) for different genotypes (**Figures 5A-C**).

This study further performed GO enrichment analysis on these DEGs. With a q-value< 0.05, the analysis (**Figures 5D-F**; **Supplementary Data Tables S4**-**S6**) categorized DEGs into BP, CC, and MF, which exhibited genotype-specific features. The WT, KN, and OE lines had 26 (1,382 DEGs), 48 (895 DEGs), and 35 (392 DEGs) enriched GO terms, respectively (**Supplementary Data Tables S4**-**S6**).

DEGs in the WT, KN, and OE lines showed partial GO enrichment overlap but also line-specific features. All three lines were involved in fatty acid metabolism and enriched in α-amylase activity (MF) and tonoplast (CC), suggesting these processes and structures play fundamental roles in their shared physiological regulation. Additionally, the protein refolding (GO:0042026) was enriched in all lines, suggesting protein homeostasis is a critical shared mechanism.

The WT group showed specific enrichment in glycolysis, carbohydrate metabolism, and various ion transmembrane transport processes, along with abscisic acid/steroid hormones responses, with single-membrane lipid storage bodies (CC) as a key cellular component. This highlights that core energy metabolism, hormone signaling, and lipid storage play pivotal role in regulating rice seed germination. In contrast, the KN group was significantly enriched in water stress response, osmotic regulation, and cell wall metabolism, as well as abscisic acid signaling and seed dormancy, with vacuolar V-type ATPase complexes (CC) notably present. The distinct enrichment suggests that *OsTIP3;1* knockout may endow rice with a unique mechanism for regulating stress responses, growth, and vacuolar function during germination. Notably, the OE group was prominently enriched in vesicle transport-related processes, with Golgi apparatus-associated structures and intracellular vesicle (both CC) being notably prominent. This enrichment pattern demonstrates that *OsTIP3;1* overexpression may specifically activate substance synthesis and intracellular transport during rice seed germination.

The GO enrichment profiles of the three groups collectively reflect the functional divergence of DEGs across distinct genetic backgrounds. These profiles not only highlight the conserved functions of core metabolic and cellular processes but also reveal group-specific molecular pathways underlying their unique characteristics in energy metabolism, biosynthesis, and stress responses.

### 3.6 KEGG enrichment analysis of DEGs

To further explore DEG-enriched pathways in the aleurone layer of WT, KN, and OE groups, KEGG pathway enrichment analysis was performed. As shown in **Figure 5G**, distinct temporal and genotypic differences in pathway enrichment were observed, with notable similarities and differences across key metabolic and regulatory pathways. Core energy and amino acid metabolic pathways, including carbon metabolism, glycolysis/gluconeogenesis, and amino acids biosynthesis were consistently enriched across all genotype comparisons (WT, KN, and OE) at multiple time points, indicating sustained regulation of energy and nitrogen metabolism to meet dynamic growth demands during germination.

For the pentose phosphate pathway, the WT and OE groups showed enrichment at 3 d vs 1 d and 5 d vs 1 d but not at 7 d vs 1 d, while the KN group exhibited the opposite pattern: lacking enrichment at 3 d vs 1 d but showing enrichment at 5 d vs 1 d and 7 d vs 1 d. Regarding the ribosome pathway, the WT and OE groups were enriched at 5 d vs 1 d and 7 d vs 1 d but not at 3 d vs 1 d, whereas the KN group showed enrichment specifically at 3 d vs 1 d and no enrichment at 5 d vs 1 d or 7 d vs 1 d. For protein processing in endoplasmic reticulum, the WT no enrichment at any time points, the KN was enriched at 5 d vs 1 d and 7 d vs 1 d, and the OE showed enrichment only at 3 d vs 1 d. Regarding fatty acid biosynthesis, the OE group showed no enrichment at 3 d vs 1 d, while the WT and KN groups were consistently enriched across all time point comparisons. For fatty acid metabolism, enriched DEGs decreased at 7 d vs 1 d in both the WT and OE groups, and at 3 d vs 1 d in the KN, while no enrichment was observed in the OE group at 3 d vs 1 d. In the fatty acid degradation, the WT and OE groups were enriched at 3 d vs 1 d, while the KN group exhibited delayed enrichment at 5 d vs 1 d.

Collectively, these results reveal that the WT maintains stable and continuous enrichment of core metabolic pathways throughout germination, with attenuation of fatty acid metabolism in late germination (7 d vs 1 d). This pattern reflects a robust, coordinated energy supply system that prioritizes foundational metabolic flux to for steady growth. In contrast, the lack of enrichment in protein processing in endoplasmic reticulum, combined with the consistent temporal patterns observed in the ribosome (enrichment at 5 d vs 1 d and 7 d vs 1 d) and pentose phosphate pathway (enrichment at 3 d vs 1 d and 5 d vs 1 d), further underscores a conservative and well-orchestrated molecular regulatory strategy. In sharp contrast, the KN group exhibits distinct reversed temporal dynamics, with ribosome enrichment at 3 d vs 1 d and pentose phosphate pathway activation at 5 d vs 1 d and 7 d vs 1 d. It shows delayed fatty acid degradation at 5 d vs 1 d, early-stage reduction in fatty acid metabolic DEGs, and late-stage enrichment in protein processing in endoplasmic reticulum. These changes suggest that *OsTIP3;1* knockout reshapes metabolic timing to prioritize early protein synthesis, stress adaptation, and late-stage protein folding/modification, likely as a compensatory mechanism for *OsTIP3;1* loss during germination. By comparison, the OE group displays unique genotype-specific pathway dynamics, lacking fatty acid biosynthesis and fatty acid metabolism at 3 d vs 1 d and showing scattered protein processing in endoplasmic reticulum enrichment only at 3 d vs 1 d. These features indicate that *OsTIP3;1* overexpression modulates lipid metabolism and metabolic flux to shift resources toward targeted substance synthesis and intracellular transport, rather than maintaining the broad, consistent metabolic activation seen in the WT.

Together, these findings highlight a shared reliance on core metabolic pathways across all genotypes while delineating striking genotype-specific temporal and functional adjustments in key pathways related to protein synthesis and lipid metabolism. These differences underscore how *OsTIP3;1* status (WT, knockout, or overexpressed) drives distinct molecular strategies for germination, providing critical insights into genotype-specific mechanisms behind their divergent metabolic responses.

### 3.7 Analysis of DEGs expression profiles

**Figure 6:**
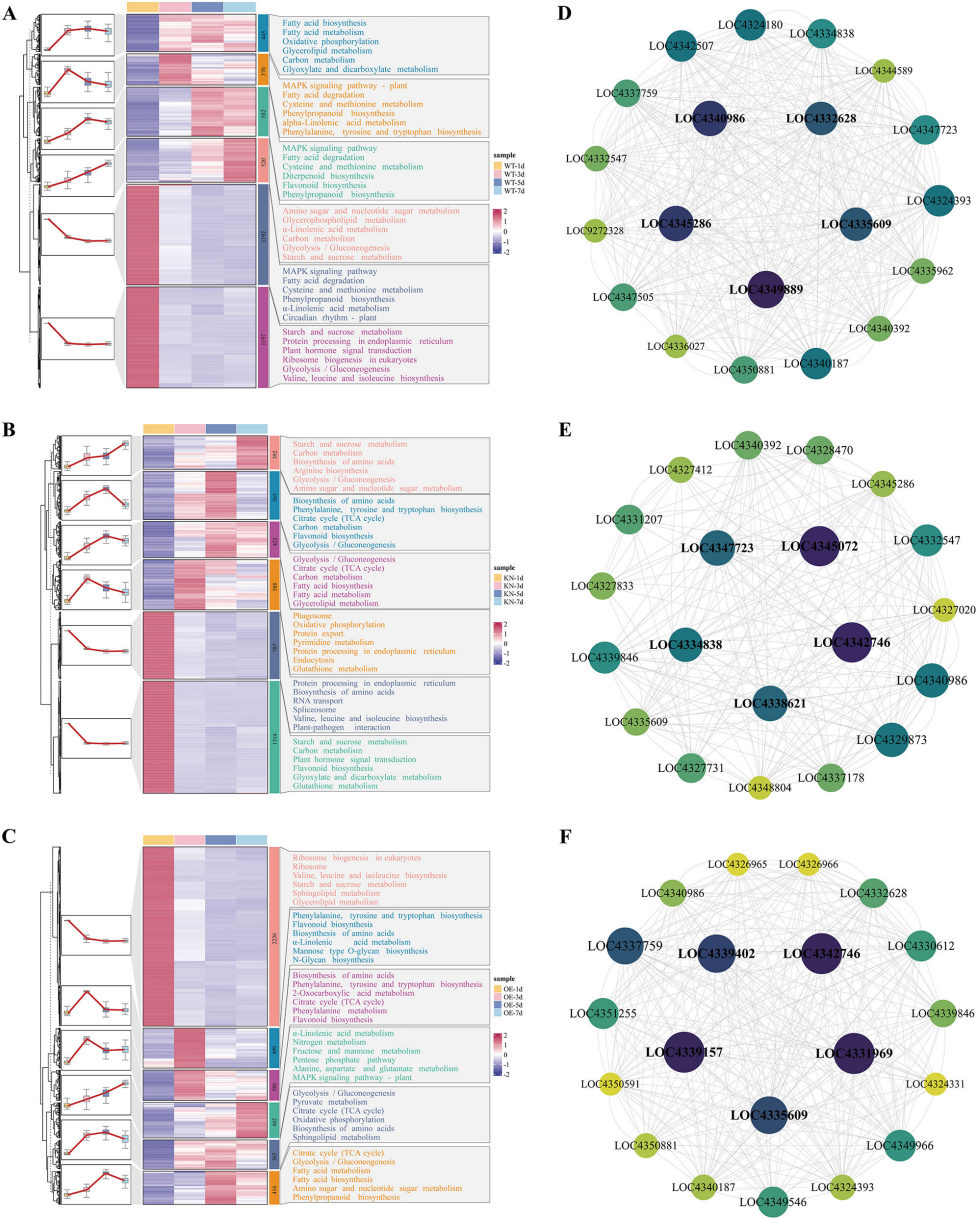
Heat maps of DEGs and protein-protein interaction (PPI) network analysis for the aleurone layer during seed germination. **(A–C)** DEGs clustering heatmaps for the aleurone during seed germination in WT **(A)**, KN **(B)**, and OE **(C)**. The color intensity indicates normalized relative gene expression levels (red = up-regulation, blue = down-regulation). **(D–F)** PPI networks of the top 20 core genes (by degree algorithm) from the WT **(D)**, KN **(E)**, and OE **(F)** lines.

To elucidate the molecular mechanism underlying *OsTIP3;1* function during rice seed germination and associate DEGs with functional KEGG pathways, we analyzed transcript profiles of shared DEGs in WT, KN, and OE lines. DEGs from each genotype were clustered into six groups using the Mfuzz algorithm (**Figures 6A-C**).

Temporal clustering of DEGs during rice seed germination reveals that *OsTIP3;1* serves as a central regulator, with its expression level orchestrating distinct metabolic and regulatory programs. In WT lines, the transcriptome reflects a balanced physiological process, coordinating core energy metabolism (e.g., oxidative phosphorylation, carbon metabolism), lipid remodeling, MAPK signaling, and the biosynthesis of amino acids and phenylpropanoids. The KN group exhibits a transcriptomic bias toward substrate mobilization and stress adaptation. In particular, the pronounced enrichment of pathways like starch/sucrose metabolism, amino acid biosynthesis, and protein processing in the endoplasmic reticulum suggests a compensatory effort to fuel germination, while the activation of stress-related pathways indicates a heightened stress response. Conversely, the OE lines display a metabolic signature geared toward prepping for subsequent growth rather than enhancing immediate growth. Correspondingly, the strong enrichment of ribosome biogenesis, coupled with active energy production (TCA cycle, glycolysis) and the biosynthesis of amino acids and structural lipids (sphingolipids), points to a metabolic program primed for rapid protein synthesis and cellular growth in later stages.

### 3.8 Screening and expression analysis of candidate gene

To investigate the molecular regulatory network of the *OsTIP3;1* gene during rice seed germination, we performed PPI network analysis on DEGs from the WT, KN, and OE lines. Using degree centrality analysis, the top 20 core genes in each line were screened, as determined by the degree algorithm. Combined with gene function annotation and inter-group characteristics, we analyzed the functions of core genes and compared their commonalities and differences among groups.

The top 20 core genes in the WT line (**Figure 6D**; **Supplementary Data Table S7**) are primarily associated with lipid transport and metabolism-related functions, including fatty acid synthesis, degradation, and energy metabolism. Genes involved in fatty acid synthesis include *LOC4335609*, which exhibits 3-oxoacyl-[acyl-carrier-protein] reductase (NADPH) activity, and participates in fatty acid chain elongation, as well as *LOC4335962* and *LOC4340392*, which possess 3-oxoacyl-[acyl-carrier-protein] synthase activity and catalyze the initiation and elongation of fatty acid chains. These genes act synergistically to lay the foundation for lipid synthesis and energy storage during rice seed germination. Genes related to fatty acid metabolism, such as *LOC4324180*, *LOC4324393*, and *LOC4340187* (long-chain acyl-CoA synthetases that act as key intermediates in fatty acid metabolism), and *LOC4332628* an enoyl-CoA hydratase involved in fatty acid degradation in peroxisomes, promote fatty acids oxidative decomposition via their isomerase and hydratase activities, thereby supplying energy for seed germination. In addition, genes like *LOC4334838* are involved in oxidative phosphorylation, linking fatty acid metabolism to energy production. Meanwhile, some genes are also associated with the biosynthesis, transport, and catabolism of secondary metabolites.

The top 20 core genes in the KN lines (**Figure 6E**; **Supplementary Data Table S7**) highlight the stress responsive regulation following *OsTIP3;1* knockout. In addition to regulating lipid synthesis and metabolism, these genes are also involved in oxidative phosphorylation via V-ATPase-related subunits. For genes encoding V-ATPase subunits, including *LOC4327412* (VHA-a1), *LOC4327731* (VHA-c’’2), *LOC4327833* (VHA-B2), *LOC4328470* (VHA-A), and *LOC4331207* (VHA-F), they act as core hubs to ensure V-ATPase function through synergistic effects in the context of *OsTIP3;1* knockout, helping cells cope with the physiological stress induced by the knockout.

In the OE lines, the top 20 core genes (**Figure 6F**; **Supplementary Data Table S7**) exhibit specific regulatory characteristics following *OsTIP3;1* overexpression, mainly involving membrane transport, substance transport, and fatty acid metabolism. Genes such as *LOC4339157*, *LOC4324331*, *LOC4331969*, *LOC4342746*, *LOC4349546*, and *LOC4351255* mediate intracellular transport, secretion, and vesicular transport, enhancing membrane transport function and substance exchange, to ensure efficient nutrients uptake and meet the demands of germination.

**Figure 7:**
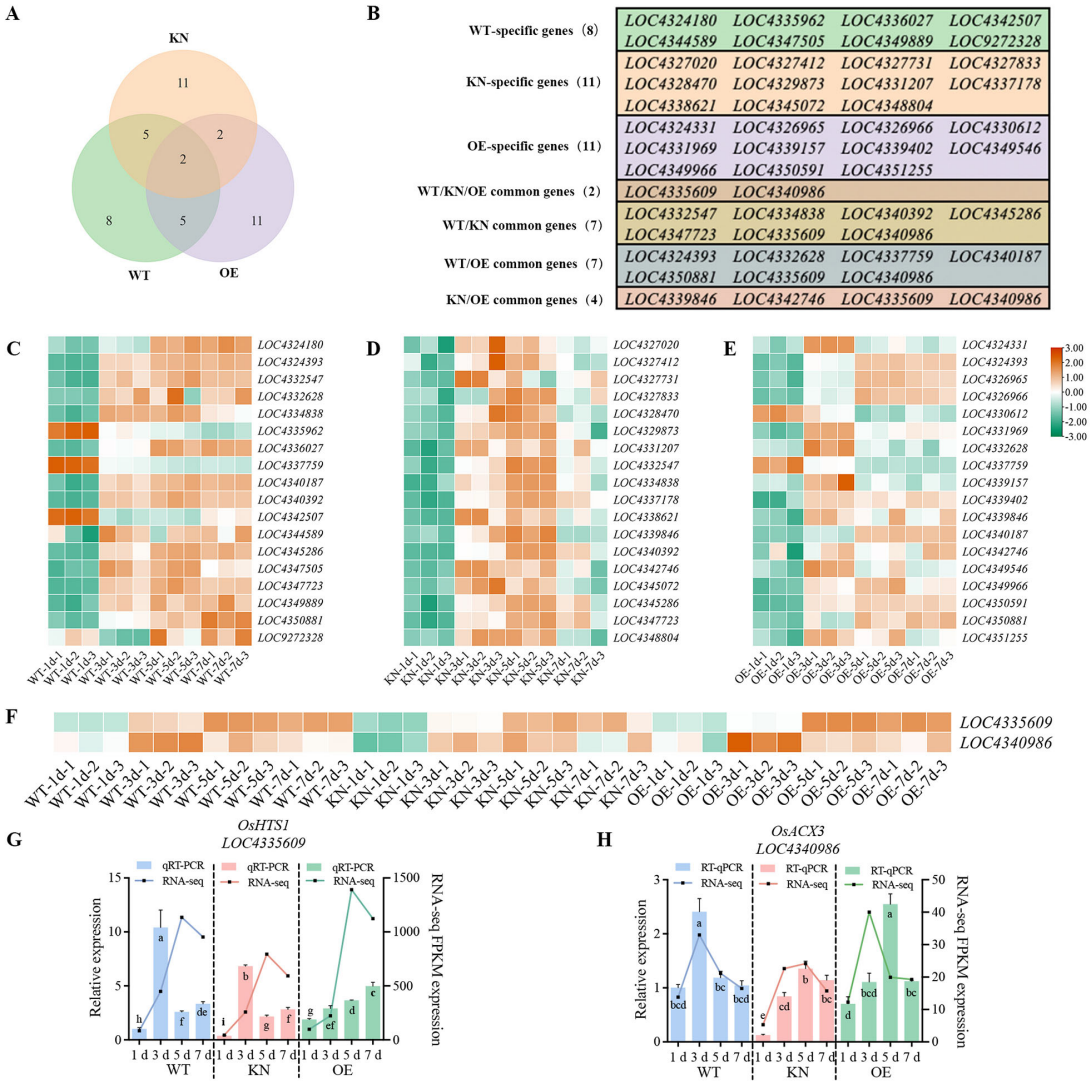
Venn diagram of core genes (top 20 by centrality) in WT, KN and OE groups. **(A)** Venn diagram of core genes in WT, KN, and OE groups, showing overlap and group-specific distributions. **(B)** List of genes in the Venn diagram, including group-specific core genes. **(C–E)** Heatmaps of core genes in WT **(C)**, KN **(D)**, OE **(E)** lines. **(F)** Heatmaps of common core genes among all three groups. All heatmaps were generated using FPKM values. **(G, H)** The expression patterns validation of *LOC4335609* and *LOC4340986* via qRT-PCR. The relative expression was calculated by the 2^⁻ΔΔCt^ method with *18S rRNA* and *Actin* as internal reference genes. Different lowercase letters in panels G and H indicate statistically significant differences at the *P* < 0.05 level; bars with the same letter are not significantly different.

Across all three groups, two genes *LOC4335609* (3-oxoacyl-[acyl-carrier-protein] reductase) and *LOC4340986* (acyl-CoA oxidase 3), serve as core hubs for lipid metabolism, regulating fatty acid chain elongation (**Figures 7A, B**). Time-course RNA-seq profiling initially demonstrated that transcript levels of both *LOC4335609* and *LOC4340986* were elevated at 3 d, 5 d, and 7 d compared to 1 d in the WT, KN, and OE lines, with these temporal expression dynamics subsequently corroborated via qRT-PCR. Notably, both RNA-seq and qRT-PCR analyses showed that, compared to the WT, both genes were downregulated at 1 and 3 d in the KN lines. For OE lines, RNA-seq data at the 3 d showed that *LOC4334609* was downregulated, while *LOC4340986* was upregulated. In contrast, qRT-PCR validation confirmed that transcript levels of both genes were decreased at 1 and 3 d in the OE lines (**Figures 7C-H**). These distinct expression dynamics suggest that the two genes may participate in *OsTIP3;1*-mediated regulation of rice seed germination through divergent roles in the lipid metabolism pathway, further supporting the notion that *OsTIP3;1* gene regulation of rice seed germination may be centered on the lipid metabolism pathway.

## 4 Discussion

### 4.1 *OsTIP3;1* ensures normal glume development and aleurone grain formation

Most studies have mainly focused on the function of TIPs as water channels in alleviating abiotic stresses like drought and salinity (Srivastava et al., 2016). TIPs also play crucial roles in developmental processes, including seed germination, radicle protrusion and cell elongation (Sudhakaran et al., 2021). In particular, the TIP3 subgroup, exhibits a conserved seed-specific expression pattern across species (Johnson et al., 1989; Novikova et al., 2014), as demonstrated in *Arabidopsis* (*OsTIP3;1*/*TIP3;2*) and barley (*HvTIP3;1*) (Gattolin et al., 2011; Utsugi et al., 2015). In rice, this pattern is reflected not only in the specific expression of *OsTIP3;1* and *OsTIP3;2* in mature seeds (Li et al., 2008) but also in their functional localization to specialized PSV in aleurone cell (Takahashi et al., 2004). Our results indicated that *OsTIP3;1* knockout caused distinct glume and mature seeds abnormalities (protruding, irregular ridges and narrowed shape of glume; ridged and concave surface of mature seeds), and abnormal aleurone grain development, while overexpression had a slight impact (**Figure 1**). This suggests that *OsTIP3;1* is required for proper glume development, possibly by regulating cell differentiation and expansion. These processes may depend on vacuolar water uptake and turgor pressure. O’Lone et al. (2024) reported that HvTIP1;1, HvTIP3;1, and HvTIP3;2 in the proteome of mature barley grain are essential for facilitating water uptake, regulating cell turgor, and promoting the large central vacuole formation to support efficient storage reserve hydrolysis and endosperm modification. They further proposed that TIP3s mediate water uptake in malting barley grain. Collectively, these lines of evidence support our findings.

### 4.2 *OsTIP3;1* controls seed germination and seedling growth

*OsTIP3;1* knockout inhibited radicle, plumule, and lateral root growth, while overexpression caused transient inhibition of radicle and plumule elongation (**Figure 3**). This suggests that *OsTIP3;1* dosage is critical for seedling growth, as both loss and excess of *OsTIP3;1* disrupt growth, though the effect of overexpression is attenuated over time. The reduced GE and GI in knockout lines indicate that *OsTIP3;1* promotes early germination uniformity, which is important for field emergence. The high final GR in all lines suggests that *OsTIP3;1* affects germination speed rather than germination capacity, possibly due to functional redundancy with other TIP family members. Candidate redundant genes (e.g., *OsTIP3;2*) are also expressed in rice seeds and may compensate for *OsTIP3;1* loss to ensure basic germination capacity (Sakurai et al., 2005; Balasaheb et al., 2020). *Zmtip3–1* mutant showed shorter shoot and root length, and decreased seedling dry weight (Su et al., 2022). In contrast, *TIP3* knockout/knockdown mutants exhibit no obvious germination and growth phenotypes but do play a role in the maintenance of seed longevity (Mao and Sun, 2015). Yeast two-hybrid assays have shown that AtSM34, which localizes in multiple tissues of *Arabidopsis* under osmotic stress, interacts with AtTIP1;1 and AtTIP2;1, an interaction that may delay germination (Li et al., 2011). Although our study has established a crucial phenotypic foundation for the role of *OsTIP3;1*, the underlying molecular mechanisms, such as its potential protein interactions, remain to be elucidate.

### 4.3 *OsTIP3;1* induces asynchronous vacuolar fusion in aleurone cells

Vacuoles are the largest, generally acidic, compartments in plant cells occupying up to 90% of the cell volume. They are classified according to their functions as PSVs and LVs. PSVs are abundant in seed and storage tissues, where they store proteins and amino acids crucial for germination and seedling development (Fluckiger et al., 2003; Nishimura and Hatsugai, 2011; Barozzi et al., 2025). The aleurone layer, a widely used model system, undergoes dramatic vacuolar remodeling during seed germination, specifically the fusion of PSVs into a large central LV (Lee et al., 2015, 2020; Betts et al., 2017). This process is not only a morphological change but a pivotal event triggering programmed cell death (PCD) and nutrient release, which is essential for germination. Previous work from our laboratory indicated that vacuole fusion in rice aleurone cells proceeds through two distinct modes, membrane fusion and embedded fusion, to form a large central vacuole. The rupture of this vacuole initiates PCD by releasing hydrolytic enzymes, disrupting plasma membrane integrity, and degrading nucleus. Importantly, the vacuole fusion inhibitor E-64d delayed formation of the large central vacuole and the subsequent PCD, which preserved numerous small PSVs, maintained cell viability, and confirmed vacuole fusion as a prerequisite for PCD execution (Zheng et al., 2017; Zhang et al., 2020).

Our results demonstrate that *OsTIP3;1* is a critical regulator of this vacuolar transition. *OsTIP3;1* knockout accelerated vacuolar fusion and rupture, while its overexpression delayed it (**Figure 2**). This establishes that *OsTIP3;1* governs the rate of vacuolar fusion, creating an asynchronous cellular phenotype within the aleurone layer, aligning with the barley model where ABA prevents PSV coalescence by strongly upregulating *HvTIP3;1* expression, while GA promotes the central vacuole formation by repressing it (Lee et al., 2015, 2020). This asynchrony provides a plausible cellular explanation for the observed germination phenotypes. Accelerated fusion in *tip3;1-ii* mutant likely leads to premature PCD and nutrient depletion, underpinning the inhibited radicle and plumule growth (**Figure 3**). Conversely, delayed fusion in OE-*tip3;1-III* lines may slow nutrient mobilization, causing the transient suppression of early seedling growth. The fact that *OsTIP3;1* affects germination speed (GI and GE) rather than GR is fully consistent with *OsTIP3;1* regulating the timing of this cellular event. Therefore, the rate of *OsTIP3;1*-mediated vacuolar fusion acts as a cellular timer, linking vacuolar dynamics to the physiological outcome of seed germination and early seedling growth. Future studies can directly validate the model by manipulating fusion rates and quantifying the phenotypic effects.

The mechanism by which *OsTIP3;1* regulates fusion timing remains unclear. Vacuolar dynamics involve complex machinery, including SNAREs, Rab GTPases, the HOPS complex, and actin microfilaments (AFs) (Brillada et al., 2018; Wu et al., 2024). Notably, AF depolymerization is required for vacuole fusion and promotes PCD, while AF stabilization inhibits both processes (Zhang et al., 2020). Therefore, we hypothesize that *OsTIP3;1*, as a tonoplast intrinsic protein, may influence the vacuolar environment interact with these core regulators (e.g., SNAREs, Rab GTPases, or components affecting AF stability) to modulate the fusion rate, though the precise mechanisms remain to be elucidated.

### 4.4 *OsTIP3;1* governs a regulatory network centered on lipid metabolism

Transcriptome analysis revealed genotype-specific DEG enrichment. For example, KN lines were enriched in stress response pathways (e.g. osmotic stress, abscisic acid signaling), OE lines in vesicular transport, and WT in core energy metabolism (**Figure 5**). Consistent with the observed phenotypes, KN lines may activate stress responses to compensate for the loss of *OsTIP3;1*, while OE lines enhance vesicular transport to support nutrient mobilization.

Lipid metabolism emerged as a key *OsTIP3;1*-regulated pathway, in which *LOC4335609* and *LOC4340986* were identified as core genes that showed significant expression changes in all genotypes (**Figure 7**). Functional annotation revealed that *LOC4335609* (*LOC_Os04g30760*, *PLS4*/*HTS1*) encodes a chloroplast-localized acyl-CoA oxidase (involved in fatty acid β-oxidation) that regulates leaf senescence, membrane integrity, and is linked to lipid metabolism in rice (Zhou et al., 2020; Chen et al., 2021). While *LOC4340986* (*LOC_Os06g24704*, *OsACX3*) encodes an acyl-CoA oxidase with developmental stage-specific β-oxidation activity, essential for seedling energy supply and fatty acid synthesis/metabolism (Kim et al., 2007; Ge et al., 2024). Notably, the latter gene colocalizes with a chromosome 6 QTL cluster associated with fatty acid content (Shen et al., 2010). Therefore, both genes are key regulators of lipid catabolism, signaling, and energy metabolism in rice. Evidently, lipid degradation in the aleurone layer supplies energy for germination (Achary and Reddy, 2021; Liang et al., 2025), and the enrichment of fatty acid metabolism in WT confirms its critical role. Unfortunately, KN lines exhibited delayed fatty acid degradation, potentially explaining their reduced seedling growth, while OE lines maintaining lipid metabolism homeostasis, consistent with growth recovery at 7 d. Collectively, our research reveals that lipid metabolism is a central pathway associated with *OsTIP3;1* function during rice seed germination, and provides new insights into the potential regulatory roles of TIPs extending beyond those previously described in *Arabidopsis* and barley. Future lipid profiling and functional validation will be critical to validate the mechanistic links proposed here.

Although lipids regulate vacuolar homotypic fusion across development stages in yeast (Starr and Fratti, 2019), evidence linking vacuolar fusion to lipid metabolism remains limited in plants. In this study, we did not verify the direct interaction between OsTIP3;1 and lipid metabolism-related genes. Furthermore. the causal relationship between asynchronous vacuolar fusion and lipid metabolism needs to be further explored.

## 5 Conclusion

In conclusion, our analysis showed that the aquaporin OsTIP3;1 plays multifaceted roles in rice seed germination, extending far beyond simple water transport. It is essential for normal seed development, ensuring proper glume formation and the structural integrity of aleurone grains. Moreover, *OsTIP3;1* critically regulates efficient germination, controlling the timing, vigor, and elongation of radicles, plumules, and lateral roots. Our study revealed that *OsTIP3;1* regulates seed germination in the aleurone layer: at the cellular level, it orchestrates the controlled fusion of intracellular vacuoles; at the metabolic level, *OsTIP3;1* coordinates the activation of lipid metabolism and other core energy pathways, thereby mobilizing nutrients to support embryonic growth.

Knockout of *OsTIP3;1* triggers transient compensatory metabolic processes in seeds, whereas its overexpression primes seeds for subsequent growth by enhancing transport capacity. Collectively, these findings advance our understanding of TIP. Thus, *OsTIP3;1* emerges as a promising target for genetic strategies to improve seed quality and germination uniformity in rice, and potentially other cereals.

## Data Availability

The data presented in the study are deposited in the National Center for Biotechnology Information (NCBI, https://www.ncbi.nlm.nih.gov/) repository, accession number PRJNA1417258.
